# Negative energy balance in a male songbird, the Abert's towhee, constrains the testicular endocrine response to luteinizing hormone stimulation

**DOI:** 10.1242/jeb.123042

**Published:** 2015-09

**Authors:** Scott Davies, Sisi Gao, Shelley Valle, Stephanie Bittner, Pierce Hutton, Simone L. Meddle, Pierre Deviche

**Affiliations:** 1 School of Life Sciences, Arizona State University, Tempe, AZ 85287, USA; 2 The Roslin Institute, The Royal (Dick) School of Veterinary Studies, The University of Edinburgh, Easter Bush, Midlothian EH25 9RG, UK

**Keywords:** Food restriction, Luteinizing hormone, Passerine, Photoperiod, Reproductive development, Testosterone

## Abstract

Energy deficiency can suppress reproductive function in vertebrates. As the orchestrator of reproductive function, endocrine activity of the hypothalamo-pituitary–gonadal (HPG) axis is potentially an important mechanism mediating such effects. Previous experiments in wild-caught birds found inconsistent relationships between energy deficiency and seasonal reproductive function, but these experiments focused on baseline HPG axis activity and none have investigated the responsiveness of this axis to endocrine stimulation. Here, we present data from an experiment in Abert's towhees, *Melozone aberti*, using gonadotropin-releasing hormone (GnRH) and luteinizing hormone (LH) challenges to investigate whether energy deficiency modulates the plasma testosterone responsiveness of the HPG axis. Wild-caught birds were either *ad libitum* fed or energetically constrained via chronic food restriction during photoinduced reproductive development. Energy deficiency did not significantly affect the development of reproductive morphology, the baseline endocrine activity of the HPG axis, or the plasma testosterone response to GnRH challenge. Energy deficiency did, however, decrease the plasma testosterone responsiveness to LH challenge. Collectively, these observations suggest that energy deficiency has direct gonadal effects consisting of a decreased responsiveness to LH stimulation. Our study, therefore, reveals a mechanism by which energy deficiency modulates reproductive function in wild birds in the absence of detectable effects on baseline HPG axis activity.

## Abstract (precis)

**Summary:** Energy deficiency in an adult male songbird has no detectable effect on the development of reproductive morphology, but constrains the endocrine responsiveness of the testes.

## INTRODUCTION

During prolonged periods of energy deficiency (i.e. when energy expenditure exceeds energy intake), animals prioritize investment of energy in the requirements for survival over investment in reproductive processes (Lack, 1954; Gittleman and Thompson, 1988; Hill et al., 2008). For example, negative energy balance can suppress reproductive cyclicity in mammals (Parfitt et al., 1991; De Souza et al., 1998; Klentrou and Plyley, 2003; Hill et al., 2008), and can also suppress reproductive function in fish (Roff, 1983), reptiles (Ford and Seigel, 1989) and birds (Hahn et al., 2005). However, the mechanisms responsible for suppression of reproductive function during times of energy deficiency in non-mammalian vertebrates are poorly understood.

Reproduction is regulated by the activity of the hypothalamo-pituitary–gonadal (HPG) axis, beginning with the release of gonadotropin-releasing hormone (GnRH) from the hypothalamus (Sharp, 2005; Zohar et al., 2010; Clarke, 2011). This neuropeptide stimulates the secretion of luteinizing hormone (LH) from the anterior pituitary gland (Kuenzel, 2000; Zohar et al., 2010; Clarke, 2011). In males, LH stimulates gonadal growth, gametogenesis, and the synthesis and secretion of testosterone (Murton and Westwood, 1977). Testosterone regulates a variety of male reproductive characteristics, such as the development of primary (Kempenaers et al., 2008) and secondary sexual characteristics (Buchanan et al., 2003), courtship, and aggression towards conspecific males (Hegner and Wingfield, 1987; Landys et al., 2010).

Food restriction of captive animals has often been used to investigate the effects of negative energetic balance (Hahn et al., 2005; Davies and Deviche, 2014). In birds, studies using this manipulation have provided evidence for a multitude of effects on the HPG axis, including the synthesis and/or secretion of GnRH (Ciccone et al., 2007), LH (Hahn, 1995; Kobayashi et al., 2002) and testosterone (Richard-Yris et al., 1987; Pérez-Rodríguez et al., 2006), and testis growth (O'Brien and Hau, 2005; Perfito et al., 2008). However, data remain sparse and are primarily drawn from domesticated species, and it is, therefore, unclear whether the results can be extended to wild birds.

In many animals, the activity of the HPG axis changes seasonally such that the gonads recrudesce at the beginning of each breeding period and regress at the end (Murton and Westwood, 1977; Silverin, 1984; Silverin et al., 1997; Dawson, 2008). Because photoperiod (day length) often forecasts environmental conditions at a given location, many seasonal breeders use the vernal increase in photoperiod as the primary cue to stimulate GnRH secretion and initiate gonadal development (Farner and Wingfield, 1980; Dawson et al., 2001; Wingfield, 2008). As changes in day length at a given latitude are constant from year to year, the activity of the HPG axis can still be modulated by non-photoperiodic cues, such as ambient temperature (Dawson, 2005; Schaper et al., 2012b) and food availability (Wingfield and Kenagy, 1986; Hahn et al., 2005; Davies and Deviche, 2014). The use of these cues is thought to improve the synchronization of reproductive development and behaviors with optimal environmental conditions in a given year (Baker, 1938; Lack, 1968; Bronson and Heideman, 1994). However, the physiological mechanisms by which non-photoperiodic cues fine-tune seasonal activity of the HPG axis remain elusive.

**List of symbols and abbreviations**

GnRH: gonadotropin-releasing hormone
GUD: giving-up density
HPG: hypothalamo-pituitary–gonadal
LD: long days
LH: luteinizing hormone
SD: short days

The measurement of baseline (pre-challenge) plasma levels of reproductive hormones can provide information about the effects of energetic balance on HPG axis activity, but these effects can be masked by high intra-individual variation in baseline hormone levels (Jawor et al., 2006; McGlothlin et al., 2010). A complementary approach to determine the functionality of the HPG axis is to measure changes in plasma testosterone in response to an endocrine challenge such as a standardized LH or GnRH injection (Deviche et al., 2012a). This approach may elucidate how negative energetic balance suppresses activity of the HPG axis for at least two reasons. First, unlike pre-challenge plasma testosterone, post-challenge plasma testosterone levels are individually repeatable (Jawor et al., 2006), potentially revealing effects of energetic status that would otherwise be masked by high intra-individual variation. Second, the plasma testosterone response to GnRH challenge parallels the seasonal change in baseline plasma testosterone (Hirschenhauser et al., 2003; Jawor et al., 2006; DeVries et al., 2011), and a range of supplementary environmental cues, such as the availability of preferred food types (Hau et al., 2000; Watts and Hahn, 2012), precipitation (Small et al., 2008a) and song (Wingfield and Wada, 1989; Small et al., 2008b), can rapidly elicit an increase in endocrine activity of the HPG axis. Therefore, the responsiveness to endocrine challenges is biologically meaningful. To our knowledge, only one other study has used this approach to examine the regulation of HPG axis activity in response to negative energetic balance in a wild-caught bird. In that study on house finches, *Haemorhous mexicanus*, food restriction constrained testicular growth and the photoinduced rise in plasma testosterone, but did not detectably influence the plasma testosterone response to GnRH or LH challenge (Valle et al., 2015). In Abert's towhees, *Melozone aberti* (Baird), negative energetic status likewise constrained the photoinduced rise in plasma testosterone, but, unlike in the house finch, this treatment had no effect on testicular growth (Davies et al., 2015a). Collectively, the findings of these two studies suggest species-specific responses of the HPG axis to energy deficiency.

Here, we used GnRH and LH challenges to investigate the effect of negative energetic balance on reproductive function in the Abert's towhee. This species is a sedentary sparrow found in riparian habitat throughout the Sonoran desert. The timing of breeding in towhees depends on precipitation and other non-photoperiodic environmental cues (Tweit and Finch, 1994). The Abert's towhee, therefore, is particularly appropriate for studies aimed at examining how non-photic factors, such as food availability, modulate activity of the HPG axis. Wild-caught male towhees held in captivity were energetically constrained via chronic food restriction. To verify that experimental birds were in negative energetic balance, we measured body mass, energy stores (as estimated by fat stores and pectoral muscle size), and the motivation of birds to forage during giving-up density (GUD) trials, which determine how depleted a patch of food must become before an animal ceases foraging (Lerman et al., 2012). The HPG axis activity of food-restricted and *ad libitum*-fed (control) birds was assessed by determining their baseline plasma LH and testosterone, as well as plasma testosterone following separate GnRH and LH challenges. We predicted that negative energetic balance would attenuate pre-challenge as well as post-challenge plasma testosterone. Finally, we tested the hypothesis that the later, but not the early, stages of reproductive development are sensitive to energetic status by measuring pre-challenge plasma LH and testosterone, and challenge-induced plasma testosterone in birds exposed to short or long day lengths.

## RESULTS

### Body mass

**Fig. 1.**
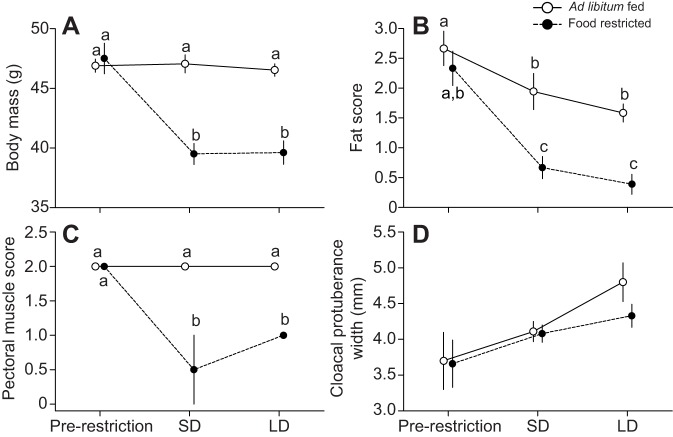
**Effect of food availability on adult male Abert's towhees, *Melozone aberti*.** Towhees were either *ad libitum* fed (*N*=9) or food restricted (70% of *ad libitum* consumption; *N*=8). Food availability modulated body mass (A), furcular fat (B) and pectoral muscle (C), but had no significant effect on cloacal protuberance width (D). The pectoral muscle graph depicts medians and half interquartile range, whereas the remaining graphs show means±s.e.m. Points with identical letters are not significantly different (*P*>0.05, Tukey's HSD test). SD, short days; LD, long days.

Body mass was affected by food availability (*F*_1,16_=15.33, *P*=0.001), time (*F*_2,32_=127.22, *P*<0.001) and the interaction between these factors (*F*_2,32_=121.50, *P*<0.001; Fig. 1). Body mass of *ad libitum*-fed birds was similar throughout the experiment (Tukey's HSD, *P*>0.05), whereas body mass of food-restricted birds decreased after 2 weeks (Tukey's HSD, *P*<0.05), and remained at the reduced level for the duration of the study.

### Fat score

Furcular fat score was affected by food availability (*F*_1,16_=10.06, *P*=0.006), time (*F*_2,32_=61.65, *P*<0.001) and the interaction between these two factors (*F*_2,32_=6.62, *P*=0.004; Fig. 1). Fat scores of birds in both treatment groups decreased over the first 2 weeks of the study, but the magnitude of the decrease was greater in food-restricted than in *ad libitum*-fed birds (Tukey's HSD, *P*<0.05). For the remainder of the study, fat scores did not change further in birds in either group (Tukey's HSD, *P*>0.05).

### Pectoral muscle score

Pectoral muscle score was affected by food availability (*F*_1,16_=138.87, *P*<0.001), time (*F*_2,32_=51.53, *P*<0.001) and the interaction between these two factors (*F*_2,32_=51.53, *P*<0.001; Fig. 1). Muscle score of *ad libitum*-fed birds was similar throughout the experiment (Tukey's HSD, *P*<0.05). Muscle score of food-restricted birds decreased after 2 weeks and remained at the reduced level for the duration of the study (Tukey's HSD, *P*>0.05).

### Cloacal protuberance width

Cloacal protuberance width increased over the duration of the study (*F*_2,32_=19.03, *P*<0.001), but was not significantly affected by food availability (*F*_1,16_=1.02, *P*=0.33) or the interaction between this factor and time (*F*_2,32_=1.13, *P*=0.34; Fig. 1). *Post hoc* analysis showed that cloacal protuberance width increased between pre-restriction and exposure to short days (SD), and again between when birds were exposed to SD and long days (LD; Tukey's HSD, *P*<0.05).

### GUD

Food-restricted birds consumed more of the food provided in the experimental trays (i.e. had lower GUDs) than *ad libitum*-fed birds (*ad libitum* fed: 0.59±0.17 g; food restricted: 1.52±0.13 g; *t*_16_=−4.34, *P*=0.0005).

### Effects of photoperiod and food availability on baseline plasma LH and testosterone

**Fig. 2.**
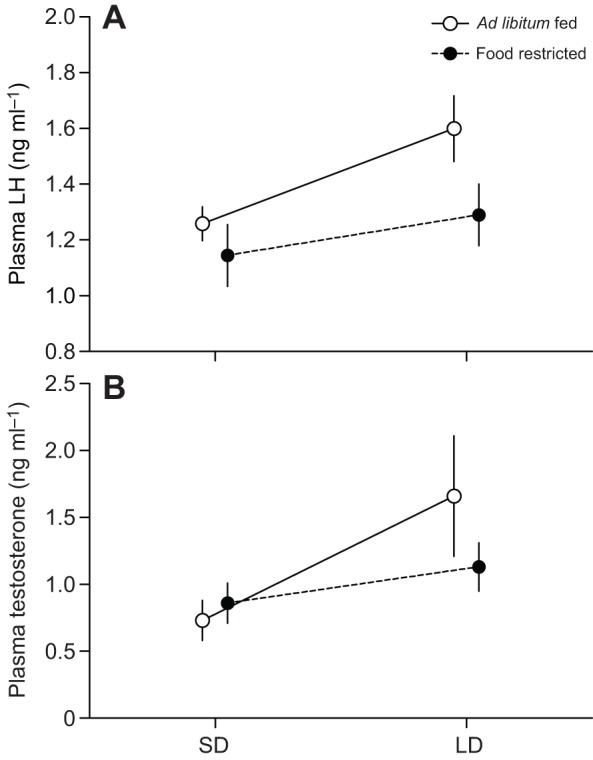
**Effect of photoperiod and food availability on pre-challenge plasma luteinizing hormone (LH) and testosterone.** Towhees were either *ad libitum* fed (*N*=9) or food restricted (70% of *ad libitum* consumption; *N*=8) and initially exposed to short day lengths before being transferred to long days. The photostimulated increase in pre-challenge plasma luteinizing hormone (A) and testosterone (B) of adult male Abert's towhees was not significantly affected by food availability. Data points are means±s.e.m.

Transfer from SD to LD increased pre-challenge plasma LH and testosterone in both *ad libitum*-fed and food-restricted birds (LH: *F*_1,16_=7.23, *P*=0.016; testosterone: *F*_1,16_=13.94, *P*=0.002; Fig. 2). However, there was neither a significant effect of food availability on pre-challenge plasma levels of these hormones (LH: *F*_1,16_=3.13, *P*=0.096; testosterone: *F*_1,16_=0.09, *P*=0.77; Fig. 2) nor a significant interaction between photoperiod and food availability (LH: *F*_1,16_=1.28, *P*=0.28; testosterone: *F*_1,16_=2.25, *P*=0.15; Fig. 2).

### Effects of GnRH challenge on plasma testosterone

**Fig. 3.**
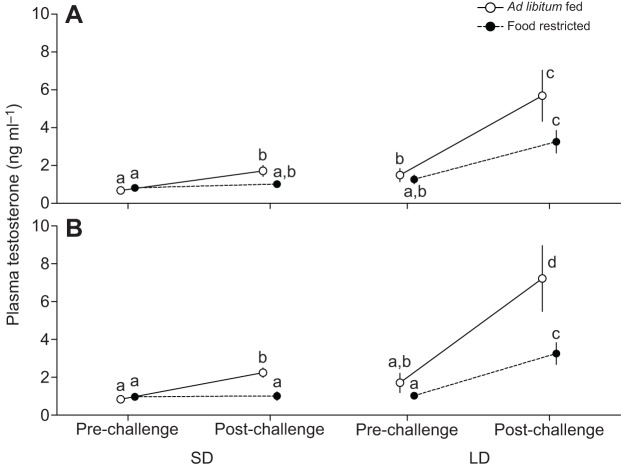
**Effect of LH and gonadotropin-releasing hormone (GnRH) challenge on plasma testosterone.** Adult male Abert's towhees were either *ad libitum* fed (*N*=9) or food restricted (70% of *ad libitum* consumption; *N*=8), and received both a GnRH challenge (A) and a LH challenge (B) while exposed to short days and again while exposed to long days. Food restriction suppressed the plasma testosterone response to a LH challenge, but there was no significant effect on a GnRH challenge. Data points are means±s.e.m., and points with identical letters are not significantly different (*P*>0.05, Tukey's HSD test).

Plasma testosterone was affected by the two-way interaction between GnRH challenge and food availability (*F*_1,16_=10.34, *P*=0.005), and GnRH challenge and photoperiod (*F*_1,16_=11.86, *P*=0.003; Fig. 3A). Under SD, GnRH challenge significantly increased plasma testosterone in *ad libitum-*fed birds, but not in food-restricted birds. Under LD, GnRH challenge increased plasma testosterone by a similar degree in both *ad libitum-*fed and food-restricted birds. GnRH challenge increased plasma testosterone in *ad libitum-*fed and food-restricted LD-exposed birds to a higher level compared with *ad libitum-*fed and food-restricted SD-exposed birds.

### Effects of LH challenge on plasma testosterone

There was an effect of the three-way interaction between photoperiod, food availability and LH challenge on plasma testosterone (*F*_1,16_=7.35, *P*=0.015; Fig. 3B). Under SD, LH challenge elicited a significant increase in plasma testosterone in *ad libitum*-fed birds, but not food-restricted birds. By contrast, LH challenge during LD increased plasma testosterone in both *ad libitum*-fed and food-restricted birds. However, the magnitude of the increase was significantly higher in *ad libitum*-fed than in food-restricted birds.

### Correlations between LH and GnRH challenge-induced change in plasma testosterone in long-day birds

**Fig. 4.**
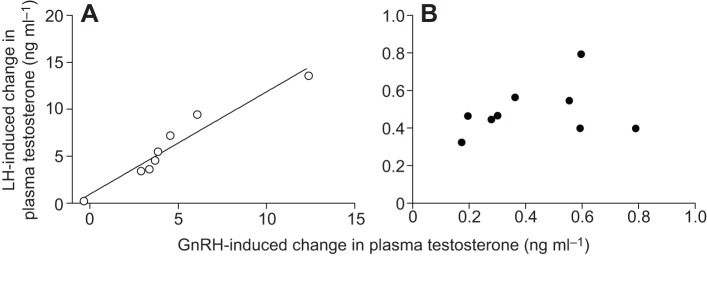
**Correlation between the effects of LH and GnRH challenges on plasma testosterone.** (A) The increase in plasma testosterone in response to GnRH challenge was correlated with the increase in plasma testosterone in response to LH challenge in long day *ad libitum*-fed adult male Abert's towhees. (B) By contrast, no such correlation was found in long day food-restricted towhees. Each point represents one individual. Note that the scales of the axes differ between the two panels.

The increase in plasma testosterone induced by GnRH challenge was correlated with the increase in plasma testosterone induced by LH challenge in LD-exposed *ad libitum*-fed birds (*R*^2^=0.94, *P*<0.0001; Fig. 4A), but not in LD-exposed food-restricted birds (*R*^2^=0.12, *P*=0.36; Fig. 4B).

## DISCUSSION

In mammals, the HPG axis is pivotal to adjust the timing of seasonal reproductive development and suppresses reproductive function during periods of energetic hardship (Bronson and Heideman, 1994; Hill et al., 2008). The mechanisms responsible for the suppression of reproductive function during negative energy balance in non-mammalian vertebrates, including birds, are poorly understood (Hahn et al., 2005; Davies and Deviche, 2014). To elucidate these mechanisms, we used chronic food restriction of captive adult male Abert's towhees to test the hypothesis that energetic status modulates plasma levels of reproductive hormones and development of reproductive morphology. Furthermore, because endocrine challenges potentially expose inter-individual differences in HPG axis function that are otherwise obscured by intra-individual variation in plasma hormone levels (Jawor et al., 2006; McGlothlin et al., 2010), we also examined whether the plasma testosterone response to GnRH or LH challenge was constrained by negative energy balance. We verified that food restriction treatment was effective in reducing body mass and energy stores (as estimated by furcular fat stores and pectoral muscle size), which indicated that food-restricted birds were in negative energetic status. We also used a behavioural assay (GUD) to test whether food restriction increased the motivation of towhees to forage from an artificial food patch. Consistent with this conclusion, food restriction decreased GUD, indicating that food-restricted birds were more motivated to forage than *ad libitum*-fed birds.

### Reproductive development and baseline endocrine activity

As day length increases during the late winter and early spring, the HPG axis of photoperiodic birds is activated, resulting in gonad development, testosterone secretion and development of testosterone-dependent secondary sexual characteristics (Dawson et al., 2001; Deviche et al., 2010). When transferred from SD to LD, towhees in the present study increased their pre-challenge plasma LH and testosterone, and developed their cloacal protuberance, the size of which in this species positively correlates with testis size (Davies et al., 2015b). These data are consistent with the hypothesis that, similar to most species studied to date, Abert's towhees are photoperiodic.

It is generally thought that the early stages of reproductive development in birds are chiefly regulated by photoperiod and are relatively insensitive to energetic status (Hahn et al., 2005), whereas the later stages of development are more sensitive to energetic status. Accordingly, most avian studies examine the effects of energetic status on the HPG axis during LD, and there are few experimental tests of whether the early stages of development are sensitive to energetic status. We, therefore, tested whether energetic status modulates HPG axis activity and development of reproductive morphology during SD as well as LD exposure. Contrary to our predictions, we found no evidence that negative energy balance caused by food restriction constrained baseline endocrine activity of the anterior pituitary gland or the testes. Likewise, we found no evidence that growth of the testes, as indicated by cloacal protuberance, was affected by energetic status.

Our finding that reproductive development was not significantly influenced by food restriction contrasts with findings from studies of domesticated avian species, in which food restriction or deprivation reduced plasma LH and follicle-stimulating hormone (Tanabe et al., 1981; Hoshino et al., 1988; Lal et al., 1990; Kobayashi and Ishii, 2002), and plasma estradiol and progesterone (Tanabe et al., 1981; Hoshino et al., 1988). Furthermore, this treatment reduced ovary and testis mass (Tanabe et al., 1981; Kobayashi et al., 2002; Ciccone et al., 2007). Our findings are, however, more consistent with studies of wild-caught birds exposed to food restriction in captivity, which have revealed conflicting relationships between the effects of this manipulation on body mass and condition, and testicular development. For example, the findings from studies in which food restriction reduced body mass are inconsistent. In both the European starling, *Sturnus vulgaris*, and the house finch, food restriction reduced photoinduced testicular growth (Dawson, 1986; Meijer, 1991; Valle et al., 2015), whereas testicular growth of Abert's towhees was unaffected by this treatment (Davies et al., 2015a). Interestingly, testicular growth in starlings was decreased only when body mass was concurrently decreased, indicating that a decrease in body mass may be necessary for food restriction to negatively affect gonadal development (Dawson, 1986; Meijer, 1991). In contrast, some studies have found effects of food limitation even when body mass and fat stores were unaffected. For example, in the garden warbler, *Sylvia borin*, and the zebra finch, *Taeniopygia guttata*, food restriction constrained testicular growth, without affecting body mass (Bauchinger et al., 2008; Perfito et al., 2008). The body mass, fat score, and testicular growth of red crossbills, *Loxia curvirostra*, were also resistant to food restriction, but this treatment delayed the photoinduced plasma LH surge (Hahn, 1995). Taken together, these observations do not indicate a consistent causal relationship between body mass, energy reserves and gonad development, and our results are consistent with this conclusion. In Abert's towhees, chronic food restriction reduced body mass and energy reserves, as estimated by fat stores and pectoral muscle mass. However, this treatment did not attenuate the stimulatory effect of LD exposure on pre-challenge plasma LH and testosterone, or cloacal protuberance width. Thus, the photoinduced reproductive development of male Abert's towhees was not detectably affected by a reduction of food availability and associated decrease in energy reserves. If the same applies to free-ranging birds, it is predicted that vernal activation of the reproductive system in male Abert's towhees is relatively independent of the food supply (mostly terrestrial arthropods; Tweit and Finch, 1994) that these birds preferentially consume. Recent work comparing free-ranging urban and rural Abert's towhees belonging to the same population as studied here support this prediction (Davies et al., 2015b). In this previous study, urban and rural habitats did not differ with respect to their terrestrial arthropod abundance, and urban and rural birds had similar body masses and fat reserves. Yet, urban males developed their reproductive system earlier in the spring than non-urban males, suggesting in these birds that factors other than food availability and body condition determine population differences in the onset of vernal gonadal development. Given the results of studies on other species described above, further research examining the relationships between food availability, body condition and photoinduced reproductive development in species with diverse life history characteristics are warranted to elucidate the bases of interspecific differences in the effects of food limitation on the vernal development of the reproductive system.

### Testicular endocrine responsiveness to hormone challenges

Besides using an increase in photoperiod to initiate gonadal development, most free-ranging birds rely on multiple non-photic factors, including food resources, to fine-tune the activity of their reproductive system and better synchronize this activity with environmental conditions (Lynn et al., 2010; Fokidis et al., 2013; Davies and Deviche, 2014). The neuroendocrine mechanisms that mediate the reproductive effects of non-photic factors remain poorly known, especially in wild birds, and may involve actions at the hypothalamus (e.g. GnRH synthesis or secretion), pituitary gland (e.g. LH production or secretion) and/or gonad (e.g. testosterone production or secretion) levels. To investigate this question, we compared the plasma testosterone response of SD- and LD-exposed *ad libitum*-fed and food-restricted birds to GnRH or LH challenge.

A GnRH challenge stimulated testosterone secretion and this effect, while more pronounced in LD- than in SD-exposed birds, did not depend on whether birds were fed *ad libitum* or food restricted. Hormone challenges have been more commonly used to assess the activity of the HPG axis in birds that are in breeding condition (Jawor et al., 2006, 2007; McGlothlin et al., 2010) than in non-breeding condition. However, a GnRH challenge also increased plasma testosterone in non-breeding northern cardinals, *Cardinalis cardinalis* (DeVries et al., 2011), and in photorefractory white-crowned sparrows, *Zonotrichia leucophrys gambelii* (Wingfield et al., 1979). These results suggest that the anterior pituitary gland and gonads retain GnRH and LH receptors, respectively, outside of the breeding season, but the affinity and/or binding capacity of these receptors may increase as the breeding season approaches (DeVries et al., 2011). A LH challenge likewise increased plasma testosterone, but, unlike a GnRH challenge, stimulated plasma testosterone more in *ad libitum*-fed than in food-restricted birds. This difference was observed whether males were photostimulated or not, but the effect of food availability on the plasma testosterone response to LH challenge was particularly pronounced in LD-exposed towhees. Indeed, *ad libitum*-fed and food-restricted LD-exposed towhees had similar plasma testosterone before LH challenge, but after this treatment plasma testosterone was more than twice as high in *ad libitum*-fed than in food-restricted birds. These results suggest that food restriction of towhees in breeding condition (i.e. photostimulated) constrains, but does not completely inhibit, the gonadal responsiveness to LH. Furthermore, we expected that the different levels of the HPG axis within a LD-exposed towhee would be synchronized such that the plasma testosterone response to a GnRH challenge and a LH challenge would be correlated. This was the case in *ad libitum*-fed towhees. However, such synchronization was abolished by food restriction. In contrast to the findings of the current study, a similar experiment in male house finches found no evidence that food restriction influences the responsiveness of the HPG axis to endocrine stimulation (Valle et al., 2015). This disparity between the findings of the two studies suggests species-specific modulation of the activity of the HPG axis during periods of energy deficiency. Alternatively or in addition, differences between the results obtained in house finches and in the present study may result from differences in experimental design and statistical analysis.

Collectively, these observations do not exclude the possibility that food restriction influenced the HPG axis at the pituitary gland level, but do demonstrate that negative energetic status caused by food restriction has direct gonadal effects consisting of decreased testicular sensitivity to the stimulatory action of LH. These findings suggest that, in male towhees, the basic pattern of photoinduced gonadal development and elevated testosterone secretion may be insensitive to food limitation and a reduction in energy reserves. However, food restriction, especially during photostimulation, may attenuate the stimulatory effects of non-photic factors that are mediated by increased LH secretion, possibly via decreases in the number of gonadal LH receptors. An alternative, but not mutually exclusive, mechanism responsible for these effects is an increase in gonadally produced gonadotropin-inhibitory hormone (GnIH), the activity of which decreases gonadal testosterone secretion and is responsive to metabolic signals (McGuire and Bentley, 2010; McGuire et al., 2013). We point out that our study was conducted on male birds, which are thought to commit less energy to reproduction than do females. We predict, therefore, that the reproductive development and gonadal endocrine responsiveness of female birds are more likely to be constrained by negative energetic status.

Constraining the testicular endocrine responsiveness to stimulation in free-ranging birds may contribute to fine-tuning reproductive behaviors to the environmental conditions in a given year. Indeed, a decreased plasma testosterone response to normally stimulatory non-photic factors would presumably also decrease the probability of food-limited birds expressing energetically costly testosterone-dependent behavior, such as singing and territory defense (Lynn et al., 2010), when energy supplies are limited and environmental conditions for successful breeding are not appropriate. In support of this proposition, spring snow storms, which likely made foraging more challenging, were associated with decreased plasma testosterone and temporary abandonment of territories by free-ranging male song sparrows, *Melospiza melodia* (Wingfield, 1985). Conversely, a return to favorable food conditions may result in male birds increasing their testicular sensitivity to LH and, therefore, also becoming more responsive to non-photic stimulatory environmental cues other than food resources. In this situation, increased testosterone secretion may enhance the expression of reproductive behavior and the development of testosterone-dependent secondary sexual characteristics (e.g. cloacal protuberance), altogether ultimately promoting an earlier onset of breeding. These changes would also be adaptive because, within limits, early seasonal breeding in many species is associated with increased reproductive success (Davies and Deviche, 2014).

In summary, using endocrine challenges, we have revealed a mechanism by which energy deficiency constrains reproductive function in wild birds. This constraint was observed in the absence of similar detectable constraints on the development of reproductive morphology or the baseline endocrine activity of the HPG axis. We suggest that this constraint on endocrine responsiveness provides a potential mechanism by which wild birds use food availability to synchronize reproductive function with optimal environmental conditions.

## MATERIALS AND METHODS

### Capture sites and captive conditions

We collected 18 adult male Abert's towhees from the Tonto National Forest, Maricopa County, AZ, USA, between 9 and 14 January 2014 (417 m above sea level; latitude: 33°32′N; longitude: 111°37′W). Birds were captured using conspecific playback and mist nets. The length of the wing chord distinguished adult males (≥92 mm) from adult females (Pyle, 1997). Upon capture, each bird received a numbered aluminium leg band and was transported to Arizona State University's Animal Care Facility where it was individually housed under a SD (10 h light:14 h dark) photoperiod in visually isolated cages (76 cm L×46 cm W×46 cm H), received *ad libitum* water and initially received *ad libitum* food (black oil sunflower seeds).

### Food restriction and experimental design

**Fig. 5.**
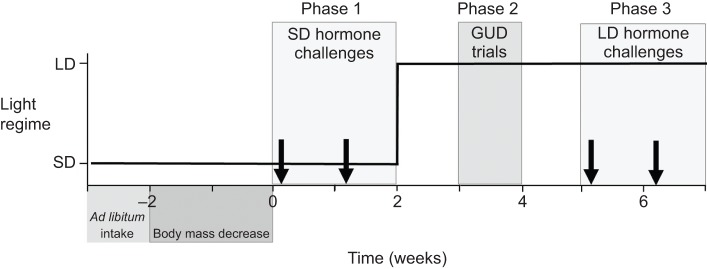
**Schematic representation of the experiment.** The change in photoperiod regime is indicated by the solid line, and the arrows show the timing of the experimental challenges. We first measured daily *ad libitum* food consumption of each bird, then food restricted half of the birds for the remainder of the experiment by giving them 70% of daily *ad libitum* consumption per day, resulting in a 15% body mass decrease. The experimental challenges began at time 0. SD hormone challenges consisted of measuring the plasma testosterone response to both GnRH and LH during short days (10 h light:14 h dark), and LD hormone challenges consisted of measuring this response during long days (16 h light:8 h dark). Giving-up-density (GUD) was measured as birds were transferred to long days.

After all birds were transferred over 7 days to a maintenance diet (Mazuri small bird maintenance diet, PMI Nutrition International, Richmond, IN, USA), the daily food consumption of each individual was monitored for 1 week (Fig. 5). This was done by providing each bird with a known amount of food and measuring the amount that remained 24 h later, both in the food bowl and on the cage floor. After this 1 week monitoring period, we randomly assigned birds to one of two treatment groups: (1) *ad libitum* food availability or (2) restricted food availability. Birds in the restricted group were given a food ration equal to 70% of their daily *ad libitum* food consumption. We selected this restriction regime based on previous studies on Abert's towhees that demonstrated that a diet of 70% of daily *ad libitum* consumption reduces body mass by approximately 15% (Davies et al., 2015a). In order to maintain a 15% reduction in body mass, we weighed birds daily to the nearest 0.5 g. If a bird's mass dropped below its target mass, we immediately fed it the difference (in grams) between its current mass and its target mass. All food-restricted birds reached their target mass after approximately 7–10 days of food restriction.

The experiment consisted of three phases (Fig. 5). In phase 1, we investigated the effects of GnRH and LH challenges (see below) on plasma testosterone while birds were exposed to SD. In phase 2, we transferred birds to LD (16 h light:8 h dark) and performed behavioral tests (GUD, see below). After 3 weeks of LD exposure, phase 3 again entailed investigating the effects of GnRH and LH challenges (see below). At the end of the experiment, all birds received *ad libitum* food for 1 week before being released at the capture site.

### Morphometrics

Before food restriction and at the time of each challenge, the amount of furcular fat was visually estimated by assigning a score of 0–5 (a score of 0 representing no fat, 5 representing bulging fat deposits; Helms and Drury, 1960), and cloacal protuberance width (±0.1 mm), an androgen-dependent secondary sexual characteristic, was measured using digital callipers. The pectoral muscles in birds are the largest store of protein, and muscle protein can be converted into energy via gluconeogenesis. The size of the pectoral muscles was estimated on a scale ranging from 0 to 3 (0 representing concave pectoral muscles and a prominent keel, 3 representing convex pectoral muscles that protruded above the keel; Salvante et al., 2007).

### Blood samples and hormone treatments

We investigated the effects of GnRH and LH challenges on plasma testosterone while birds were exposed to SD (10 February 2014; phase 1) and then again when they were exposed to LD (phase 3; see below). For this, we collected a blood sample (200 µl) from the right jugular vein into a heparinized microsyringe within 3 min of removing a bird from its home cage. Less than 3 min later, we gave one randomly assigned intrajugular injection of either synthetic GnRH-I (Sigma Chemical Co., MO, USA; 25 µg kg^−1^ body mass) or freshly prepared ovine LH solution (The National Peptide and Hormone Program, Torrance, CA, USA; 1 mg kg^−1^ body mass) dissolved in 100 µl of sterile saline solution (0.9% NaCl). Birds were then placed into individual breathable cloth bags and bled again (200 µl) 20 min later. The injection volumes, hormone concentrations, and times between injection and blood sample are similar to those of previous studies on passerines, which have successfully used these treatments to assess HPG functionality (Jawor et al., 2006; McGlothlin et al., 2010; Deviche et al., 2012a). Blood samples were placed on ice immediately after collection and centrifuged within 1 h. Plasma was harvested and stored at −80°C until assayed for LH and testosterone. One week later (17 February 2014), these procedures were repeated, but each bird received the opposite hormone treatment from that of the first week.

To determine the endocrine and morphological effects of LD exposure and to compare the effectiveness of GnRH and LH challenges in stimulating plasma testosterone in SD- and LD-exposed birds, the above procedure was repeated 1 month later (10 and 17 March 2014), after the birds had been exposed to LD for 3 weeks.

### GUD trials

Between phases 1 and 3 of the main experiment, we conducted a series of GUD trials. GUD examines how depleted a patch of food must become before an animal ceases foraging (Abu Baker and Brown, 2009; Lerman et al., 2012) and can be used to estimate hunger and the motivation to forage. We created a homogeneous sand–food mixture using dry sand that was pre-strained of large grains using a wire mesh strainer. We thoroughly mixed the strained sand with the maintenance diet at a food:sand mass ratio equal to 1:150, which had previously been used to quantify GUD in free-ranging Abert's towhees (Lerman et al., 2012).

We filled clear plastic trays (29 cm L×18 cm W×13 cm H) with approximately 4 cm of the sand–food mixture. Other studies on Abert's towhees and other bird species used a similar depth of sand (Abu Baker and Brown, 2009; Lerman et al., 2012). Lerman et al. (2012) used 24 h trials to quantify GUD of free-ranging Abert's towhees, but these authors did not investigate captive, food-restricted birds. Out of concern that the food-restricted birds might consume all the food provided in their tray, we first tested the effects of 2, 4, 6 or 8 h-long trials. Residual food was present in the trays at all time points, but little food was eaten after 2 or 4 h. Therefore, for the GUD trials, we selected the intermediate time of 6 h.

In order to train the birds to feed from the plastic trays, during the first week of LD exposure all birds were provided with sand-filled trays scattered with a small amount of food for at least 24 h. During the 2 days of GUD trials, we placed the filled trays in the birds’ cages at 10:00 h each day. Birds also received their normal food ration as described above. We collected the food remaining in each tray 6 h later by separating it from the sand using a wire mesh strainer, and then weighed it to the nearest milligram. The mass (g) of remaining food was used to calculate GUD.

### Hormone assays

To quantify plasma LH, we used the radioimmunoassay described by Sharp et al. (1987) with slight modifications. This radioimmunoassay has been used to determine plasma LH in a many bird species (Lal et al., 1990; Lea et al., 1991; Malecki et al., 1998; Ciccone et al., 2007; Schaper et al., 2012a; Fraley et al., 2013), including multiple emberizid sparrows (Meddle et al., 2002; Deviche et al., 2008; Wingfield et al., 2012; Deviche et al., 2012a,b). Briefly, the reaction volume of 60 µl was composed of 20 µl of plasma sample or standard, 20 µl of primary rabbit LH antibody and 20 µl of I^125^-labeled purified chicken LH. The primary antibody was precipitated to separate free and bound I^125^ label using 20 µl of donkey anti-rabbit precipitating serum and 20 µl of non-immune rabbit serum. All samples were assayed in duplicate and in a single assay, for which the intra-assay coefficient of variation was 3.6% and the minimum detectable concentration was 0.2 ng ml^−1^. The post-GnRH challenge samples could not be assayed for LH.

We measured plasma testosterone using a validated (Davies and Deviche, 2015) commercial enzyme-linked immunoassay following the manufacturer's recommendations (Enzo Life Sciences, Farmingdale, NY, USA). Plasma was diluted 10× in assay buffer containing 1 µl displacement reagent:99 µl plasma. Each of the four assay plates included a complete standard curve. Samples were assayed in duplicate in one assay and randomly assigned to plates, except that all the samples from a given towhee were assayed on the same plate. The assay sensitivity was 18.1 pg ml^−1^ and the inter- and intra-assay coefficients of variation were 3.2% (*N*=3 samples assayed on each plate) and 6.7% (*N*=154 samples), respectively. The primary antibody used in this assay has less than 5% cross-reactivity with 17β-estradiol, 5α-dihydrotestosterone, corticosterone and progesterone (manufacturer's specifications).

### Statistical analysis

The raw data from the study are presented in supplementary material Table S1. We statistically analyzed data using SPSS 21 (SPSS Inc., Chicago, IL, USA) and SigmaPlot 12.5 (Systat Software, Inc., San Jose, CA, USA) with α=0.05. We used repeated measures ANOVA (rmANOVA) to test whether food availability affected body mass, furcular fat score, pectoral muscle score, cloacal protuberance width, and pre-challenge plasma LH and testosterone over the course of the study. For each of these variables, we used the average of the two measurements taken from a given bird during each phase of the experiment. To meet the assumptions of normality and equal variance, pectoral muscle score was ranked (Conover and Iman, 1981) and plasma LH and cloacal protuberance width were log-transformed before analysis. The effect of food availability on GUD was tested using a Student's *t*-test. The effect of food availability on the plasma testosterone response to GnRH and LH challenges was analyzed with rmANOVA with day length (SD versus LD) and challenge (pre- versus post-challenge) as within-subject factors. To meet the assumptions of normality and equal variance, all plasma testosterone data were first log-transformed. Where appropriate, we followed all ANOVA with Tukey's HSD tests for pairwise multiple comparisons. We used linear regression to test for an association between the change in plasma testosterone in response to a challenge of GnRH versus LH. Data are presented as untransformed means±s.e.m.

We were unable to collect pre-challenge and post-challenge blood samples from two birds, resulting in 4 out of 72 samples missing from the data set. We estimated missing plasma LH and testosterone values using multiple imputation (MI) and the NORM program (http://sites.stat.psu.edu/~jls/misoftwa.html; Schafer, 1999). As pointed out by other authors (Hill et al., 2003), MI is more appropriate than standard approaches (e.g. case deletion or replacement of missing values by group means) because it relies on a more plausible assumption (Little and Rubin, 2002), it properly accounts for uncertainty about missing values (leading to appropriate standard errors) and it retains original sample sizes.

## Supplementary Material

Supplementary Material
