# Supplementary material for: Negative energy balance in a male songbird, the Abert's towhee, constrains the testicular endocrine response to luteinizing hormone stimulation
Source: J Exp Biol. 2015 Sep;218(17):2685–93. doi: 10.1242/jeb.123042 (PMC4582157; doi:10.1242/jeb.123042)
Supplement: Supplementary Material [file supp_218_17_2685__index.html]

Supplementary Material 

# Negative energy balance in a male songbird, the Abert's towhee, constrains the testicular endocrine response to luteinizing hormone stimulation

## JEB123042 Supplementary Material

- Supplementary Material
