# Supplementary material for: Negative energy balance in a male songbird, the Abert's towhee, constrains the testicular endocrine response to luteinizing hormone stimulation
Source: J Exp Biol. 2015 Sep;218(17):2685–93. doi: 10.1242/jeb.123042 (PMC4582157; doi:10.1242/jeb.123042)
Supplement: Supplementary Material [file supp_218.17.2685_JEB123042supp.pdf]

Table S1

Click here to Download Table S1
